# Nutritional assessment, phytochemical composition and antioxidant analysis of the pulp and seed of medjool date grown in Mexico

**DOI:** 10.7717/peerj.6821

**Published:** 2019-07-17

**Authors:** Ricardo Salomón-Torres, Noé Ortiz-Uribe, Benjamín Valdez-Salas, Navor Rosas-González, Conrado García-González, Daniel Chávez, Iván Córdova-Guerrero, Laura Díaz-Rubio, María del Pilar Haro-Vázquez, José Luis Mijangos-Montiel, Antonio Morales-Maza, Padmanabhan Mahadevan, Robert Krueger

**Affiliations:** 1 Departamento de Posgrado, Universidad Estatal de Sonora, San Luis Rio Colorado, Sonora, México; 2 Laboratorio de Materiales Avanzados, Universidad Autónoma de Baja California, Mexicali, Baja California, México; 3 Laboratorio de Procesos Industriales, Universidad Autónoma de Baja California, Mexicali, Baja California, México; 4 Centro de Graduados e Investigación en Química, Instituto Tecnológico de Tijuana, Tijuana, Baja California, México; 5 Facultad de Ciencias Químicas e Ingeniería, Universidad Autónoma de Baja California, Tijuana, Baja California, Mexico; 6 Instituto Nacional de Investigaciones Forestales, Agrícolas y Pecuarias, Mexicali, Baja California, México; 7 University of Tampa, Tampa, FL, United States of America; 8 National Clonal Germplasm Repository for Citrus and Dates, USDA-ARS, Riverside, CA, United States of America

**Keywords:** Date palm, *Phoenix dactylifera* L., Medjool, Fatty acid, Antioxidant activities, Chemical composition, Minerals, Seed date, Mexico

## Abstract

The aim of this study was the characterization of fatty acids, antioxidant activity, some physical properties, nutrient content, sugars, and minerals in the pulp and seeds of the date cultivar ‘Medjool’ (*Phoenix dactylifera* L.) grown in Mexico. The samples were obtained at maturity (Tamar) in the 2017 harvest season in the valleys of San Luis Rio Colorado and Mexicali, Mexico. The following average values were obtained on a % dry weight basis for pulp and seeds, respectively: protein, 3.14% and 4.84%; lipids, 0.75% and 9.94%; fiber, 6.34% and 66.79%; total sugars, 75.32% and 5.88%; reducing sugars, 70.26% and 4.40%; and sucrose, 5.06% and 1.46%. Analysis of the minerals revealed that the most abundant elements for the pulp were: potassium, 851.98 mg/100 g; magnesium, 142.97 mg/100 g; and phosphorus, 139.40 mg/100 g, whereas for the seeds, they were potassium, 413.36 mg/100 g; sulfur, 151.36 mg/100 g; and phosphorus, 92.42 mg/100 g. Gas chromatography-mass spectrometry analysis revealed that the major unsaturated fatty acid was oleic acid, at 52.34% and 45.92%, respectively, for pulp and seeds. The main saturated fatty acids were palmitic acid (6.75%) and lauric acid (17.24%) in pulp and seeds, respectively. The total phenolic content was 1.16 and 13.73 mg GAE/100 g for pulp and seeds, respectively. Finally, the antioxidant activities were: b-carotene, 65.50% and 47.75%; DPPH, 0.079 IC_50_ g/L and 0.0046 IC_50_ g/L; and ABTS, 13.72 IC_50_ g/L and 0.238 IC_50_ g/L, respectively. The results obtained in this study confirm that the ‘Medjool’ cultivar grown in Mexico has the same quality of nutrients and antioxidants as those grown in the other main date-producing countries.

## Introduction

The commercial cultivation of the date palm (*Phoenix dactylifera* L.) in Mexico has developed in the northwest part of the country during recent decades. Although the Mexican date industry is very small compared to those of the large date-producing countries, date production in Mexico has increased up to 600% during the last 35 years (Ortiz-Uribe, Salomón-Torres & Krueger, 2019).

The ‘Medjool’ cultivar was introduced to the American continent at the beginning of the 20th century in Southern California in the United States (Krueger, 2015). At the end of the 1960s, the ‘Medjool’ was brought to the San Luis Rio Colorado Valley in the State of Sonora and later into the Mexicali Valley in the State of Baja California, becoming the main date cultivar grown in Mexico (Ortiz-Uribe, Salomón-Torres & Krueger, 2019). These two valleys accounted for 97% of date production in Mexico for the year 2017 (SIAP, 2019). Currently, approximately 94% of date production in Mexico is ‘Medjool’, the remainder being comprised of other cultivars, such as ‘Deglet Noor’, ‘Khadrawy’, ‘Zahidi’, ‘Bahree’, ‘Honey’, and ‘Hallawy’, with a small production of *criollo* dates (Ortiz-Uribe, Salomón-Torres & Krueger, 2019). The ‘Medjool’ cultivar has distinct advantages over other cultivars, such as high yields, high quality fruits, high value in the international market, and high nutritional value.

The area planted in dates, particularly the ‘Medjool’ variety, has increased greatly in recent years both in Mexico (Ortiz-Uribe, Salomón-Torres & Krueger, 2019) and the United States (Krueger, 2015). Trees in recent plantings are young and have not reached full production in many cases. As the trees come into full production, the overall production of dates produced in North America will increase. Due to culture and tradition, date consumption in both countries is historically low compared to that in Middle Eastern and North African countries (Krueger, 2015; Ortiz-Uribe, Salomón-Torres & Krueger, 2019). Therefore, increased production of dates may depress prices unless additional consumption of dates or new markets for them are developed. Additional domestic consumption of dates in both countries may be stimulated in part by emphasizing the health and nutritional benefits, as is being done in the United States (California Date Commission, 2019). Development of export markets depends on producing a high quality date, since production costs are higher in North America than in other date-producing countries. Therefore, it is important to verify that the physical characteristics and nutritional value of dates produced in North America are consistent with those associated with the specific varieties, and that their consumption may have beneficial effects on human health. Dates have been produced commercially in the United States for over a century and these factors have been documented. However, commercial date production in Mexico is a relatively recent development, and documentation of the characteristics and nutritional value of Mexican-produced dates is needed.

The objectives of this study were to characterize the physico-chemical components, evaluate the fatty acid profile, and quantify the antioxidant activity from the pulp and seed of ‘Medjool’ cultivated under the agroclimatic conditions of northwestern Mexico in order to evaluate their quality compared to those grown in other countries. Our working hypothesis is that the quality of the ‘Medjool’ cultivar grown in Mexico is equal to that in the main date producing countries.

## Materials & Methods

### Characterization of the experimental area

**Figure 1:**
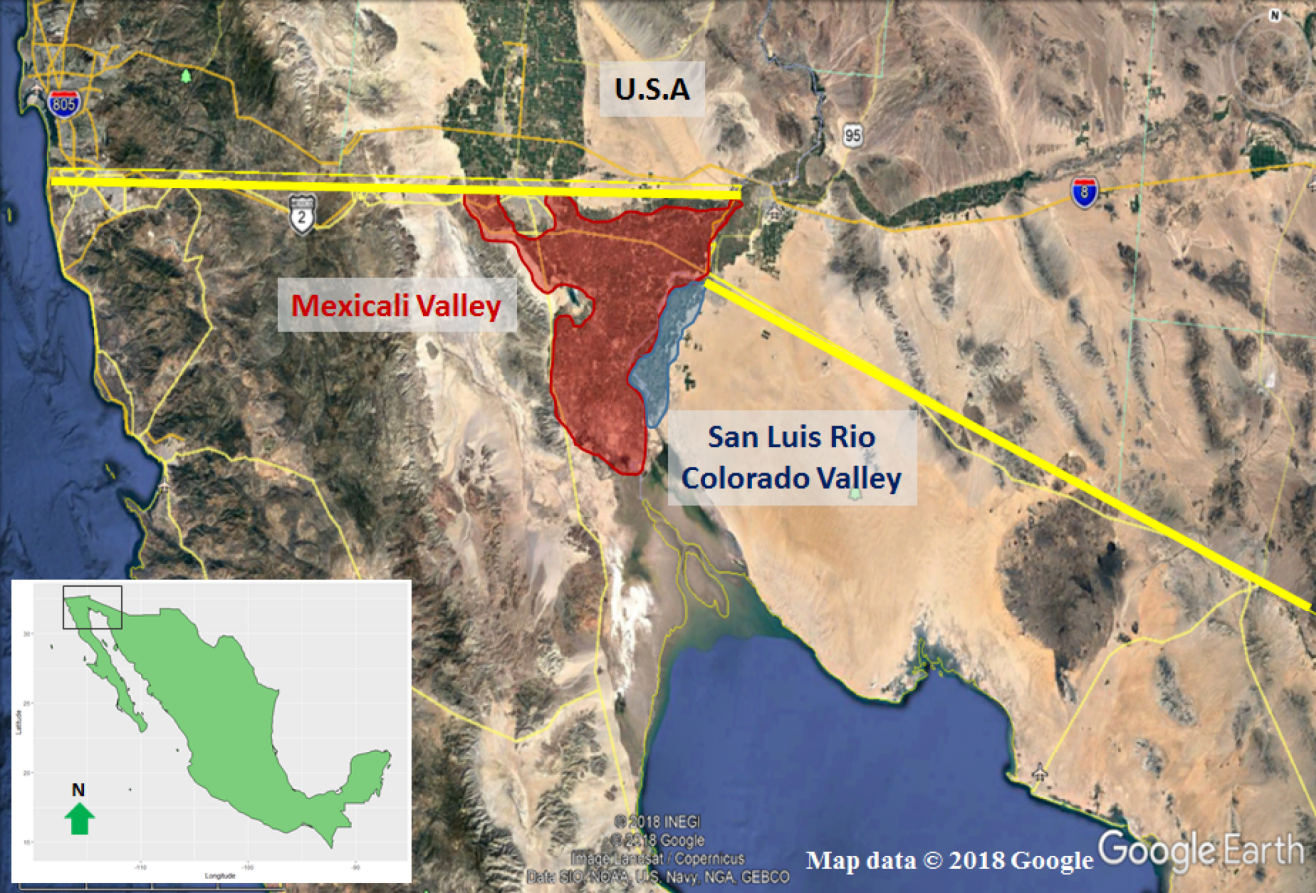
The largest areas of date cultivation in México are found in the Valleys of San Luis Rio Colorado, Sonora (area in blue) and Mexicali, Baja California (area in red).

This study was carried out during the 2017 growing season in two certified organic plantations (Fig. 1). The first was located in the Valley of San Luis Rio Colorado (32°23′5″N, 114°53′55″W) and the second in the Valley of Mexicali (32°31′17″N, 115°4′17″W). Flood irrigation was employed in an alluvial, organically amended soil. Rafael Quirarte-Gutiérrez and Roberto Torres-Yescas from Corporativo RUVA provided verbal permission to access field sities, and Dalila González-Machado and Carlos Zambrano-Reyes from SADER Baja California, provided all the information available in Mexico of this crop.

### Plant materials and pollination

**Figure 2:**
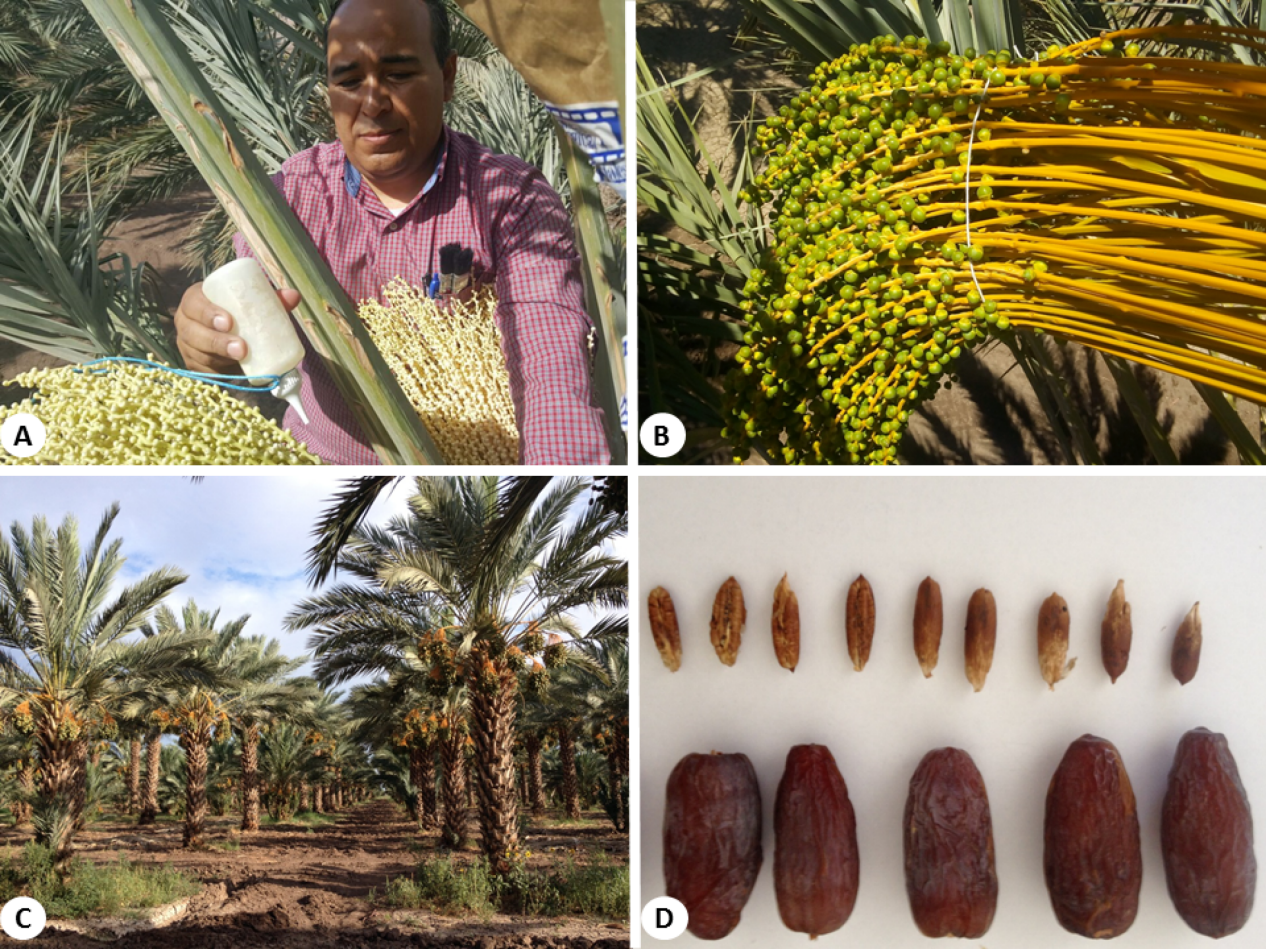
(A) Manual pollination process using a plastic squeeze bottle. (B) View of bunch 45 days after pollination. (C) Panoramic view of a Medjool date plantation in Ejido Jiquilpan, in the Mexicali Valley, México. (D) Fruit harvested in the middle of the Tamar stag.

Female plants were derived from a vigorous 16-year old ‘Medjool’ palm. Palms were planted in an 8 × 8 m pattern. Pollen was extracted from male palms of the most common cultivars in the area (‘Medjool’, ‘Deglet Noor’, ‘Khadrawy’, and ‘Zahidi’). Pollen from all sources was mixed at a 1:1 ratio with commercial wheat flour and pollination was carried out manually using a plastic squeeze bottle between the third and seventh day after the spathe naturally broke (Fig. 2).

### Treatments and sample collection

Thinning was done approximately 45 days after pollination. For each palm, 12–15 bunches were left, each being thinned to approximately 50 strands per bunch and 12–14 dates per strand. Collection of fully mature fruit samples was carried out in both valleys in the middle of the harvest season (Tamar stage) at the end of September, 2017.

### Physical properties

100 samples from each location were randomly selected and individually weighed using an analytical balance. The seed was removed and the pulp and seed were weighed separately. The average weight of the fruit, fruit pulp, seed, percentage of pulp, and ratio fruit/seed were calculated. Other physical properties such as length and diameter of the fruit and seed were expressed in centimeters (Rahnama & Rahkhodaei, 2014).

### Proximate analysis

Samples from the previous analysis, consisting in five dates and three replicates of each were analyzed. The moisture content was determined after drying 5 g of fresh weight from each sample at 105 °C for 24 h. The ash percentage was calculated by weight differences (Association of Official Analytical Chemists (AOAC), 2005), with the samples incinerated at 550 °C for 8 h in a muffle oven. Total nitrogen was determined through a chemical digestion (Kjeldahl technique), and protein was estimated using the general factor of 6.25 (University of Nebraska, 2019). Total lipids were determined according to the method of (Folch, Lees & Stanley, 1957). Total insoluble and soluble solids were determined using °Brix (Ruck, 1969). The total acidity was calculated titrating against 0.1 N NaOH and was expressed as malic acid percentage. The pH was taken from a homogeneous sample at 20 °C using a MP 744 pH meter (Metrohm AG).

### Sugars determination

Another five samples were taken and three replicates of each were used. Total sugars and reducing sugars were calculated using the Lane and Eynon volumetric method (Oberoi & Sogi, 2017). Non-reducing sugar (sucrose) was calculated as the difference between total sugars and reducing sugars. Fructose and glucose were determined using an enzymatic glucose analyzer (Galant, Kaufman & Wilson, 2015).

### Minerals analysis

For the analysis of minerals, five samples and three replicates of each were analyzed. The mineral content was determined using standard methods (Association of Official Analytical Chemists (AOAC), 2005). Around 2 g of pulp and seed from each sample was converted to ash at 550 °C for a 24 h period. One g of ash was dissolved in 5 mL of analytical grade hydrochloric acid (20%) and the solution was transferred to a 50 ml volumetric flask. The final volume was completed using deionized water. The total minerals were measured by atomic absorption spectrophotometer (model 4200 MP-AES; Agilent Technologies) and were expressed in mg/100 g dry weight basis.

### Fatty acid analysis

Extraction of seed oil from date seeds was performed using a Soxhlet extractor according to the method described by Association of Official Analytical Chemists (AOAC) (2005). 10 g of date powder was extracted with 200 mL of n-hexane in a Soxhlet extractor for 6 h. The n-hexane was removed by evaporation using a rotary evaporator at 80 °C in a water bath. The extracted oil was kept in the bottle and stored at −20 °C until use. Oil yield was expressed as the percent of oil obtained based on the weight of date powder used.

The evaluation of fatty acids in date seeds requires the preparation of fatty acid methyl esters (FAME) in order to improve volatility and to reduce peak tailing, allowing analysis by Gas Chromatography with good precision and reproducibility (Cert et al., 2000). The AOCS method Ce 2-66 was used for the preparation of FAME (American Oil Chemists’ Society (AOCS), 1997).

The FAME preparations were analyzed by Gas Chromatography/Mass Spectrometry (GC/MS) and were performed for qualitative and quantitative analysis of the phytochemicals present in the extract. The analytical GC/MS system used was an Agilent 7890A GC coupled to 5975C Mass detector (Agilent Technologies) equipped with a HP-5MS capillary column (30 m × 0.25 mm × 0.25 µm) (Agilent Technologies, Inc). An Agilent Technologies 7693 autosampler was used to inject 1 µL of a solution sample. The ionization energy was 70 eV with a mass range of 30–800 m/z. The initial temperature of the column at 125 °C, held for 0.5 min, ramp 25 °C/min to 150 °C, held for 2 min, then up to 200 °C with a 50 °C/min rate. The temperature of the injector was set at 255 °C and the detector to 270 °C. The flow rate of the carrier gas (Helium) was 1.0 mL/min injected with a gas dilution of 1:50. Identification of the individual components was based on comparison with the mass spectra library (NIST98). All samples were run in triplicate.

### Scanning electron microscopy analysis

**Figure 3:**
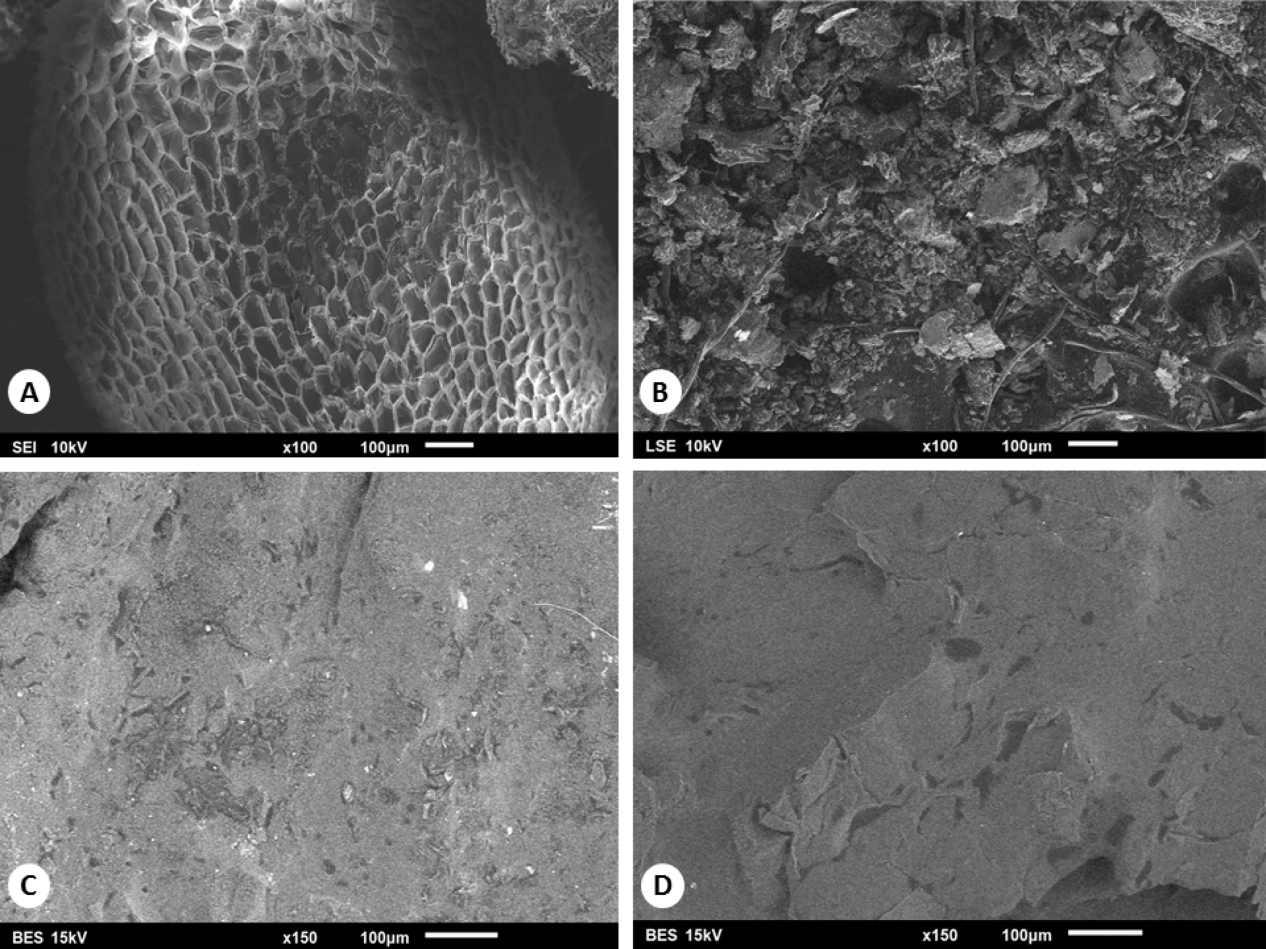
Micrographs of the external surface of date pulp and seed, previous (A, C) and after oil extraction with solvent (B, D).

The pulp and seed were analyzed using a scanning electron microscope JEOL JSM-6360 to identify the surface morphology before and after oil extraction. The conditions were constant in both images (Fig. 3).

### Total phenols and antioxidant activities

Total phenolics in date extracts were determined colorimetrically by using Folin–Ciocalteu reagent described by Perez-Meseguer et al. (2016) with slight modifications and results were expressed as milligrams equivalent of gallic acid per 100 g dry weight (mg GAE/100 g, dw). The free radical scavenging activity of pulp and seed extracts was determined by three methods: oxidation of β-carotene and linoleic acid system (Burda & Oleszek, 2001), DPPH radical scavenging activity (Salazar-Aranda et al., 2015), and ABTS radical cation decolorization assay (Re et al., 1999; Kuskoski et al., 2005). Results were expressed as an IC_**50**_ (DPPH and ABTS assay) and percentage (β-carotene assay).

### Qualitative phytochemical screening

The pulp and seed extracts were subjected to qualitative chemical tests to identify the presence of chemical constituents present using the following methods: (1) alkaloids: Meyer, Wagner, and Dragendorff reagents; (2) sterols and triterpenes: Liebermann/Burchard reagent; (3) tannins: ferric chloride and gelatin; (4) saponins: Bubble test; (5) flavonoids: Shinoda test; (6) coumarins: Erlich reagent; (7) anthraquinone glucosides: alkaline reaction; (8) carbohydrates: Benedict’s reagent (Cai, Shi & Gao, 2011; Tiwari et al., 2011; Romo-Asunción et al., 2016).

### Energy value determination

The energy value for pulp and seed was calculated using the content of protein, fat, and carbohydrates (total sugars): Energy value (kcal/100 g) = (2.62 × % protein) + (8.37 ×% fat) + (4.2 × % carbohydrates) (Crisan & Sands, 1978).

### Statistical analysis

Data analysis was performed using Microsoft Excel software. All the results were expressed as mean values ± standard deviation of the three separate determinations by sample.

## Results

### Physical characteristics of fruit, pulp, and seeds

Table 1 presents the average physical characteristics of the fruit and seed of date cultivar ‘Medjool’ grown in Mexico. The weight, length, and average diameter of the fruit were 22.12 g, 5.04 cm, and 2.6 cm, respectively. Studies conducted in Iran and Jordan reported 19 g and 35.4 g for the weight, 4.76 cm and 5.17 cm for the length, and 2.71 cm and 3.58 cm for the diameter, respectively (Ghnaim & Al-Muhtaseb, 2006; Rahnama & Rahkhodaei, 2014). The average weight of the pulp in our study was 20.82 g, representing 94.12% of the total weight of the fruit, which is similar to the results obtained in Jordan, with a pulp percentage of 94.5% (Ghnaim & Al-Muhtaseb, 2006). In the case of the seeds, weight, length, and diameter were 1.33 g, 2.59 cm, and 0.88 cm, respectively. In the United Arab Emirates, measurements of 1.1 g, 2.09 cm, and 0.93 cm, respectively, were reported for the same parameters ‘Medjool’ seeds (Habib & Ibrahim, 2009).

**Table 1:** Physical characteristics of fruit and seed of date cultivar ‘Medjool’ grown in Mexico.

**Component**	**Fruit**	**Seed**
Weight (g)	22.12 ± 3.02	1.33 ± 0.08
Length (cm)	5.04 ± 0.20	2.59 ± 0.11
Diameter (cm)	2.6 ± 0.13	0.88 ± 0.03
Length/diameter (cm)	1.93	2.94

**Notes.**

Values are mean ± SD of 200 samples, expressed on fresh weight basis.

### Proximate composition of pulp and seed

The proximate composition of pulp and seed are summarized in Table 2, revealing a moisture content of 25.81%, protein content of 3.14%, lipid content of 0.75%, fiber content of 6.34% and ash content of 2.62% (dw) for the pulp. These results are in general agreement with those reported by Bouhlali et al. (2017a) and Bouhlali et al. (2017b) in Morocco, where they reported moisture content of 30.82%, protein content of 3.45%, lipid content of 0.31%, and ash content of 2.30% (dw). They did not report the fiber content. Likewise, the results of the present study contrast with those reported by Vinita & Punia (2016) in India, where they reported moisture content of 85.42%, protein content of 1.88%, lipid content of 0.17%, fiber content of 1.96%, and ash content of 3.02% (dw), for the same cultivar. For the case of the seed, the values obtained in the present study were: moisture content 2.06%, protein content 4.84%, lipid content 9.94%, fiber content 66.79%, and ash content 1.24% (dw). These results contrast with those reported by Bouhlali et al. (2017b), who reported: moisture content 8.25%, protein content 6.14%, lipid content 5.66%, fiber content 19.90%, and ash content 1.09% (dw).

**Table 2:** Proximate composition of date pulp and seed of date cultivar ‘Medjool’ grown in Mexico (%, dw).

**Component**	**Pulp**	**Seed**
Moisture^a^	25.81 ± 1.43	2.06 ± 0.65
Protein^b^	3.14 ± 0.21	4.84 ± 0.42
Lipids^b^	0.75 ± 0.05	9.94 ± 0.08
Fiber^b^	6.34 ± 1.47	66.79 ± 3.50
Ash^b^	2.62 ± 0.12	1.24 ± 0.04
Total carbohydrates	67.67%	81.92%

**Notes.**

Values are mean ± SD of five samples with three independent determinations.

a Expressed on fresh weight basis.

b Expressed on dry weight basis.

The percentages of total soluble solids, insoluble solids, and total acidity in the pulp were 68.24%, 15.70%, and 0.08% (% dw), respectively, and pH was 6.86 (Table 3). For the seed, the following values were obtained: total soluble solids 5.19%, insoluble solids 96.84%, total acidity 0.05% (dw) and pH 6.98 (Table 3). No studies were found that described these same parameters for the ‘Medjool’ cultivar grown in other countries.

**Table 3:** The content of total soluble and insoluble solids, total acidity (% dry weight), and pH of pulp and seed of date cultivar ‘Medjool’ grown in Mexico.

**Component**	**Pulp**	**Seed**
Total soluble solids	68.24 ± 4.27	5.19 ± 0.15
Insoluble solids	15.70 ± 1.57	96.84 ± 2.01
Total acidity	0.08 ± 0.01	0.05 ± 0.01
pH	6.86 ± 0.05	6.98 ± 0.04

**Notes.**

Values are mean ± SD of five samples with three independent determinations.

### Sugars content analysis

Date pulp is characterized by its high content of sugars as shown in Table 4. The sugar contents of the pulp were: total sugars 75.32%, reducing sugars 70.265%, sucrose 5.06%, glucose 37.21%, fructose 33.17% (dw); energy content was 330.83 Kcal/100 g. These results are in general agreement with the study developed in India (Vinita & Punia, 2016), which reported total sugars 67.49%, reducing sugars 61.31%, and non-reducing sugars (sucrose) 6.18% (dw) for the ‘Medjool’ cultivar. In Morocco, Bouhlali et al. (2017a) reported an average of 33.96% and 37.79% (dw), for glucose and fructose, respectively, and 313.05 Kcal/100 g of energetic value. Higher values were obtained from Mexican-grown ‘Medjool’ for total sugars, reducing sugars, and glucose. However, for non-reducing sugars and fructose lower values were found in Mexican-grown ‘Medjool’, compared those grown in in India and Morocco.

**Table 4:** Sugar content of pulp and seeds of date cultivar ‘Medjool’ grown in Mexico (% dry weight).

**Component**	**Pulp**	**Seed**
Total sugars	75.32 ± 2.91	5.86 ± 0.20
Reducing sugars	70.26 ± 2.98	4.40 ± 0.05
Sucrose	5.06 ± 0.07	1.46 ± 0.15
Glucose	37.21 ± 1.89	–
Fructose	33.17 ± 1.50	–
Energetic value^a^	330.83	120.48

**Notes.**

Values are mean ± SD of five samples with three independent determinations.

a Expressed in Kcal/100 g.

Likewise, for the seed, total sugars were 5.86%, reducing sugars were 4.40%, sucrose was 1.46% (dw), and energy content was 120.48 Kcal/100 g. Glucose and fructose were not measured the ‘Medjool’ seed. In Morocco (Bouhlali et al., 2017b), 9.54% (dw) was reported for total sugars, contrasting with the result reported by this study. No other studies were found that reported the rest of the parameters seeds of the ‘Medjool’ cultivar.

### Mineral content analysis

The mineral content of pulp and seed of ‘Medjool’ is shown in Table 5. The most abundant mineral elements found in the pulp were: potassium 851.98, magnesium 142.97, phosphorus 139.40, calcium 129.14, and sulfur 109.89 (mg/100 g, dw). The contents of potassium (849.58), zinc (0.37), and manganese (0.32) (mg/100 g, dw) obtained in a study in Morocco (Bouhlali et al., 2017a) are in close agreement with our results. On the other hand, iron content of Mexican-grown ‘Medjool’ was very low (0.31 mg/100 g dw) in comparison the content (1.14 mg/100 g, dw) obtained in Morocco (Bouhlali et al., 2017a). In contrast, the results obtained for calcium (54.2), magnesium (67.78), sodium (11.21), and copper (0.34) (mg/100 g, dw), were lower in Morocco (Bouhlali et al., 2017a) than in our study. The rest of the minerals reported in Table 5 in pulp were not reported by Bouhlali et al. (2017a).

**Table 5:** Mineral content for date pulp and seed of ‘Medjool’ cultivar grown in Mexico (mg/100 g, dry weight).

**Component**	**Pulp**	**Seed**
Potassium	851.98 ± 21.29	413.36 ± 13.3
Magnesium	142.97 ± 6.09	35.95 ± 3.2
Calcium	129.14 ± 4.20	54.22 ± 4.5
Phosphorus	139.40 ± 3.98	92.42 ± 5.7
Sulfur	109.89 ± 1.39	151.36 ± 12.5
Sodium	27.78 ± 0.26	34.07 ± 3.9
Silicon	11.21 ± 3.43	0.79 ± 0.1
Selenium	5.39 ± 0.41	4.06 ± 0.2
Copper	1.03 ± 0.13	0.83 ± 0.1
Iron	0.31 ± 0.02	1.32 ± 0.2
Manganese	0.44 ± 0.02	0.76 ± 0.1
Zinc	0.26 ± 0.07	1.08 ± 0.3

**Notes.**

Values are mean ± SD of three independent determinations.

In the seed, the most abundant elements were: potassium (413.36), sulfur (151.36), phosphorus (92.42), calcium (54.22), and magnesium (35.95) (mg/100 g, dw). The mineral results contrast with those reported by Shi et al. (2014), who report the lower contents for potassium (146.5), calcium (22.7), and sodium (15.42) but higher results for magnesium (74.92), phosphorus (146.5), and iron (5.05) (mg/100 g, dw). The rest of the minerals in the seed shown in Table 5 were not reported by Shi et al. (2014).

### Fatty acid composition

The characterization of the fatty acid composition of the pulp and seed of date Medjool cultivar is shown in Table 6. The date pulp oil showed seven fatty acids present, of which three were 11.38% saturated, three were 58.06% mono-unsaturated, and one was 30.56% poly-unsaturated. The most abundant fatty acids were oleic acid (52.34%), linoleic acid (30.56%), palmitic acid (6.75%), vaccenic acid (4.8%), and stearic acid (3.98%). No other studies were found that report the fatty acid profile for the pulp of the ‘Medjool’ date.

**Table 6:** Fatty acid composition of pulp and seed of ‘Medjool’ dates grown in Mexico (%).

**Component**	**Pulp**	**Seed**
Caprylic (C8:0)	–	0.27 ± 0.01
Capric (C10:0)	–	0.36 ± 0.01
Lauric (C12:0)	–	17.24 ± 0.19
Myristic (C14:0)	–	10.72 ± 0.24
Palmitic (C16:0)	6.75 ± 0.04	10.76 ± 0.13
Palmitoleic (C16:1)	–	0.07 ± 0.001
Margaric (C17:0)	–	0.06 ± 0.002
Stearic (C18:0)	3.98 ± 0.04	4.79 ± 0.12
Oleic (C18:1)	52.34 ± 0.30	45.92 ± 0.68
Vaccenic (C18:1)	4.8 ± 0.55	–
Linoleic (C18:2)	30.56 ± 0.41	9.06 ± 0.01
Eicosanoic (C20:0)	0.65 ± 0.01	0.45 ± 0.02
Eicosenoic (C20:1)	–	0.21 ± 0.02
Gondoic (C20:1)	0.92 ± 0.03	–
TOTAL	100	99.91

**Notes.**

Values are mean ± SD of three independent determinations.

For the seed, twelve fatty acids were present, eight of which were saturated, representing 44.65% of the composition, while three were 46.2% mono-unsaturated and one was 9.06% polyunsaturated. The most abundant fatty acids were oleic (45.92%), lauric (17.24%), palmitic (10.76%), myristic (10.72%), and linoleic (9.06%). These results were in agreement with Bouhlali et al. (2017b), who reported 44.92%, 20.39%, 9.82%, 11.82%, and 8.47%, for the same fatty acids respectively, for ‘Medjool’ seeds.

### Scanning electron microscopy results

Micrographs of pulp and seed, obtained by Scanning Electron Microscopy (SEM), were taken before and after the oil extraction process (Fig. 3). Figure 3A shows the internal heterogeneous cellular structure, whereas Fig. 3B shows the amorphous structure after the oil extraction by solvent. Date pulp imaged before oil extraction at the tamar stage (Fig. 3C) shows the cells already destroyed, showing only some granular structures that disappear after the oil extraction with solvent (Fig. 3D).

### Antioxidant activity and phytochemical screening

Total polyphenolic content and antioxidant activities of the pulp and seed of the ‘Medjool’ cultivar are shown in the Table 7. The polyphenolic content was 1.16 (mg GAE/100 g, dw) for the pulp, while the seed showed 13.73 (mg GAE/100 g, dw). These results are in accordance with other studies (Bouhlali et al., 2017a; Bouhlali et al., 2017b).

**Table 7:** Total phenolic content and antioxidant activities of ‘Medjool’ dates grown in Mexico.

**Component**	**Total phenolic content (mg GAE/100 g, DW)**	β-Carotene (%)	DPPH (IC_50_**g/L**)	ABTS (IC_50_**g/L**)
Pulp	1.16 ± 0.006	65.50 ± 5.47	0.079 ± 0.014	13.72 ± 0.647
Seed	13.73 ± 0.68	47.75 ± 7.20	0.0046 ± 0.002	0.238 ± 0.045
Quercetin^a^	N/A	N/A	0.003 ± 0.00025	0.05 ± 0.0017
α-tocopherol^a^	N/A	60 ± 5.5	N/A	N/A

**Notes.**

Values are mean ± SD of five samples with three independent determinations.

a Used as a positive control.

N/A not apply

Antioxidant activity was analyzed with three techniques. DPPH results gave an IC_**50**_ of 0.079 and 0.0046 g/L for the pulp and seed, respectively. Bouhlali et al. (2017a) and Bouhlali et al. (2017b) reported an EC_**50**_ of 5.25 g/L for date pulp and 0.133 g/L for seed, showing a lesser degree of antioxidant activity than in our study. In the present work, we expanded the analysis further by including a positive control, the flavonoid quercetin, which presents an IC_**50**_ of 0.003 g/L in the DPPH assay.

The results for the ABTS assay for the pulp and seed were an IC_**50**_ of 13.72 g/L and 0.238 g/L, respectively. Quercetin as a positive control in this antioxidant technique had an IC_**50**_ of 0.05 g/L. Results for the ABTS assay were not as close to the control as we had in the DPPH assay.

The results for the β-carotene oxidative decolorization assay were 65.5% of antioxidant activity for the pulp and 47.75% for the seed, while the positive control (α-tocopherol) showed 60% of activity, less than shown by the pulp. It is important to mention that no studies were found reporting the antioxidant activity in the pulp and seed of ‘Medjool’ dates employing the β-carotene technique.

The results of the qualitative analysis of metabolites in the pulp and seeds of ‘Medjool’ dates grown in Mexico are shown in Table 8. The phytochemical screening results were positive for tannins, triterpenes, steroids, and carbohydrates, while negative for alkaloids, flavonoids, saponins, anthraquinones, glucosides, and coumarins. In the case of the seed, all the parameters were positive except alkaloids, triterpenes, and steroids.

**Table 8:** Phytochemical screening in pulp and seed of ‘Medjool’ dates grown in Mexico.

**Metabolites**	**Pulp**	**Seed**
Alkaloids	−	−
Tannins	+	+
Triterpenes and steroids	+	−
Flavonoids	−	+
Saponins	−	+
Carbohydrates	+	+
Anthraquinones and glucosides	−	+
Coumarins	−	+

**Notes.**

+ Detected − Not detected

## Discussion

In general, our results (Table 1) were similar to the physical characteristics of the fruit and seed of ‘Medjool’ reported in studies carried out in the United Arab Emirates, Jordan, and Iran (Ghnaim & Al-Muhtaseb, 2006; Habib & Ibrahim, 2009; Rahnama & Rahkhodaei, 2014). The existing differences may be influenced by the soil, water, and climate in the rich alluvial soils of the San Luis and Mexicali valleys. These characteristics may also be influenced by cultural practices such as thinning treatments on the strands, the number of strands per bunch, and pollen source. The identification of these characteristics of the fruit is very important for farmers since they define the quality of the fruit as well as the classification of its commercial value (Salomon-Torres et al., 2017).

Several studies of the date cultivars grown commercially in various countries have been carried out to determine their chemical and nutritional properties (Mohamed et al., 2014; Assirey, 2015; Vinita & Punia, 2016; Bouhlali et al., 2017a). In these studies, the most studied parameters were the chemical composition, sugar content, mineral content, and antioxidant activity. Some of the characteristics of date fruits are their high levels of sugars, minerals, and vitamins. The most abundant element in the pulp is sugar, with content that can vary between 70% and 80% (g/100 g), followed by fiber content (6.40–11.50%), protein (2.30–5.60%), lipids (0.20–0.50%), and minerals (4.9–1,088) (mg/100 g). Dates are a good source of phenols, carotenoids, and flavonoids. They also contain high levels of essential amino acids and vitamins such as A, B1, B2, B3, and C, as well as strong antioxidant, anticancer and antiviral activities (Assirey, 2015). Al-Farsi & Lee (2008) made a quantitative comparison of the chemical composition of the date against other nuts, such as plum, apricot, figs, peaches and raisins, concluding that the date is low in moisture and fat, as well as a rich source of carbohydrates, sugars, fiber, vitamin C, carotenoids, phenols and antioxidants, compared to the other fruits.

The pulp of ‘Medjool’ dates grown in Mexico contained low amounts of protein, lipids, and fiber (Table 2). However, the amounts of these elements in its seeds were higher. The comparative differences of the proximal analysis from this study, as compared to the ‘Medjool’ date cultivated in Morocco and India, could have been influenced by different environmental and growth conditions, affecting the composition of both the pulp and the seed. Protein is one the most important nutrients in food, but the date is not a good source, since its protein content is low. The dietary fiber content of dates makes them ideal for the preparation of foods rich in fiber and dietary supplements, as 100 g of date fruits provide 32% of the daily intake of dietary fiber (Vinita & Punia, 2016). The moisture content of the fruit affects its quality and influences its storage. It has been reported that the optimal humidity content for storage is 24–25% (Falade & Abbo, 2007). The moisture content of Mexican-grown ‘Medjool’ fruit in this study was 25.81% (dw) (Table 2), which is near optimal.

Carbohydrates are the most abundant component in dates and are present as reducing sugars (glucose, fructose, mannose, and maltose) and non-reducing sugars (mainly sucrose), with small amounts of polysaccharides such as cellulose and starch (Al-shahib & Marshall, 2003). The date is an excellent source of energy due to its high content of easily digestible sugars, such as glucose and fructose (Bouhlali et al., 2017a), providing 290 kcal/100 g (Al-Farsi & Lee, 2008). High sugar contents were found in the pulp of ‘Medjool’ dates grown in Mexico (Table 4). The most abundant sugars were glucose, fructose, and in lower proportion, sucrose. This ratio between reducing and non-reducing sugars is common in most soft varieties of dates. The sugar content of seeds was very low compared to the pulp (Table 4). Since the seeds are not consumed, their composition is not directly relevant to health or dietary needs.

The fruit and seeds of Mexican-grown ‘Medjool’ dates were an excellent source of macro- and micro-minerals (Table 5). Macro-minerals are essential for the formation and proper functioning of bones, teeth, muscles, soft tissue, hemoglobin, and nerve cells (Al-Farsi & Lee, 2008). The combination of high potassium content and low sodium can help prevent or control hypertension, while the consumption of magnesium and calcium supports vascular function, maintains proper body growth and facilitates mobility of the bones (Bouhlali et al., 2017a). Micro-minerals are required in small amounts in our body. Elements such as copper, iron, zinc, and manganese play an essential role in metabolic pathways. Iron is essential for red cell production and high potassium and low sodium are suitable for people with hypertension (Assirey, 2015). In the present study, the most abundant mineral in the pulp and seed was potassium. Likewise, the following five most abundant minerals in the pulp and seed were magnesium, calcium, phosphorus, sulfur, and sodium, but in a different order and proportion in the pulp and seed. Sodium is not mentioned in other studies, but its content in pulp and seed was higher than copper, iron, manganese, and zinc in this study (Table 5). The differences found compared to the comparative studies (Ghnaim & Al-Muhtaseb, 2006; Habib & Ibrahim, 2009; Rahnama & Rahkhodaei, 2014; Vinita & Punia, 2016) could be due to the type of soil and the nutrition of the crop, since in our case, both fields had organic certification.

The content of lipids in the seeds of Mexican-grown ‘Medjool’ dates was close to 10%, while in the pulp it was only 0.75%, (dw) (Table 2). The low percentage of lipids in the pulp indicates that the extraction of oil is not viable due to the low content of fatty acids. However, because the seed does not have an agricultural use, the commercial use of date seed oil could be exploited in the industry.

A study carried out in common date varieties grown in Tunisia determined that fatty acids contained in the pulp are composed of 67% unsaturated and 32% saturated fatty acids whereas fatty acids in the seed consisted of 59% unsaturated and 40% saturated fatty acids (Saafi et al., 2008). Commercial varieties, such as ‘Medjool’ and ‘Deglet Noor’, have been reported to contain between 55% and 59% unsaturated fatty acids and 30% to 44% saturated fatty acids (Shi et al., 2014; Bouhlali et al., 2017b). Of the fatty acids found in ‘Medjool’ date seeds in the present study, saturated fatty acids accounted for 44.65%, monosaturated fatty acids accounted for 46.2%, and polyunsaturated fatty acids accounted for 9.06% (Table 6). These proportions are similar to those reported in the referenced papers. Unsaturated fats are generally considered more benign to human health than saturated fats. ‘Medjool’ seed oil had relatively lower saturated fat levels compared to coconut oil, but higher saturated fat levels compared to olives (El et al., 2018). The most abundant fatty acid in the ‘Medjool’ seeds studied was oleic with 45.92%, which is higher than the percentage found in coconut oil (5.2%), but lower than in olive oil (71.71%) (Nasir et al., 2018).

There are numerous reports about the high concentration of phenolic acids in the date, and specifically a higher concentration in the seed, giving it a great antioxidant potential (Sundar et al., 2017). As our results show (Table 7), the total polyphenolic content in the seed of ‘Medjool’ grown in Mexico was almost ten times the quantity of the pulp. These results are similar to previous reports. The antioxidant potential of date seeds is mostly attributed to the presence of phenols (principally acids like the p-coumaric, ferulic and sinapic), flavonoids, and vitamins (Saafi et al., 2011). In this study, we observed a high total polyphenolic content for the seed (13.73 mg GAE/100 g, dw) and the presence of flavonoids was confirmed with the Shinoda assay (Table 8). Dietary polyphenolics from date consumption may supply substantial antioxidants which, in turn, may provide health promoting and disease preventing effects (Tohidi, Rahimmalek & Arzani, 2017).

The antioxidant activity, analyzed with the DPPH technique, showed better IC_**50**_ results in our study than reported by previous works (Bouhlali et al., 2017a; Bouhlali et al., 2017b), although it can be noticed that the seed extracts have better antiradical capacity over the pulp in ours and other studies. Using quercetin as a positive control, the seed extract in this study showed the closest alignment of DPPH data to the quercetin control.

Quercetin was used also as positive control in the ABTS assay. Although the seed again had a better activity compared with the pulp, neither extract approached the control. The β-carotene oxidative decolorization required a different positive control, this being α-tocopherol. Despite the antioxidant activity associated with this compound, the pulp had greater β-carotene concentration. This as the only antioxidant technique in which the pulp showed better results than the seed. Nevertheless, the seed extract, which showed a better antioxidant profile (DPPH and ABTS), had the higher total polyphenolic content.

## Conclusions

The ‘Medjool’ date cultivar is a great source of carbohydrates and essential elements. In this study, the physical and phytochemical properties of ‘Medjool’ grown in Mexico were quantified, as well as their profile of fatty acids and their antioxidant activity. The results obtained suggest that their consumption can play an important role in nutrition and human health, since they are a rich source of minerals and antioxidants. They are a great source of energy, due to their high sugar content in the pulp. This could be used as a substitute for sugar in the food industry. Also, the seed provides a rich source of fatty acids, which could serve as a basis for the development of products in the cosmetics, pharmaceutical, and food industries. This study confirms that date cultivar ‘Medjool’ grown in Mexico has the same or better nutrients and antioxidant properties as in the main date producing countries. These results provide reference information for the most important date variety grown in Mexico.

##  Supplemental Information

10.7717/peerj.6821/supp-1 Dataset S1 Raw data

## Additional Information and Declarations

### Field Study Permissions

The following information was supplied relating to field study approvals (i.e., approving body and any reference numbers): Rafael Quirarte-Gutiérrez and Roberto Torres-Yescas from Corporativo RUVA provided verbal permission to access field sites.

### Data Availability

The following information was supplied regarding data availability: The raw data is available in Supplemental File.
