# Supplementary material for: Nutritional assessment, phytochemical composition and antioxidant analysis of the pulp and seed of medjool date grown in Mexico
Source: PeerJ. 2019 Jul 17;7:e6821. doi: 10.7717/peerj.6821 (PMC6648623; doi:10.7717/peerj.6821)
Supplement: Dataset S1 [file peerj-07-6821-s001.docx]

Raw data to Table 1: Physical characteristic of date fruit Medjool cultivar.

| Component | R1 | R2 | R3 | R4 | R5 | R6 | R7 | R8 | R9 | R10 | R11 | R12 | R12 | R4 | R15 | R16 | R17 | R18 | R19 | R20 |
| --- | --- | --- | --- | --- | --- | --- | --- | --- | --- | --- | --- | --- | --- | --- | --- | --- | --- | --- | --- | --- |
| Weight (g) | 20.00 | 20.50 | 26.80 | 21.80 | 20.80 | 20.90 | 25.10 | 21.10 | 19.20 | 20.00 | 20.10 | 20.20 | 25.00 | 20.60 | 20.00 | 23.60 | 21.30 | 23.50 | 25.64 | 20.15 |
|  | 24.70 | 23.10 | 24.21 | 24.80 | 23.35 | 22.50 | 25.30 | 25.00 | 25.49 | 22.70 | 19.50 | 19.30 | 21.50 | 20.90 | 24.60 | 22.90 | 19.40 | 18.10 | 19.70 | 18.40 |
|  | 24.60 | 27.70 | 25.23 | 28.10 | 27.40 | 25.70 | 26.20 | 24.70 | 25.40 | 27.50 | 27.40 | 24.90 | 21.00 | 25.40 | 28.00 | 27.50 | 25.80 | 26.90 | 26.20 | 28.10 |
|  | 27.10 | 26.40 | 25.50 | 28.20 | 27.70 | 27.80 | 27.70 | 24.60 | 22.90 | 29.10 | 25.90 | 25.65 | 22.60 | 25.30 | 28.20 | 27.90 | 26.50 | 25.80 | 26.80 | 25.30 |
|  | 21.12 | 18.50 | 19.50 | 20.10 | 20.58 | 21.30 | 18.20 | 19.70 | 20.90 | 20.10 | 22.50 | 21.36 | 21.63 | 20.50 | 20.20 | 20.80 | 22.12 | 23.70 | 22.20 | 20.98 |
|  | 18.50 | 18.20 | 22.20 | 21.40 | 17.10 | 18.20 | 19.50 | 18.60 | 18.50 | 21.30 | 18.10 | 19.60 | 19.10 | 19.00 | 19.50 | 18.60 | 20.45 | 19.50 | 19.90 | 20.30 |
|  | 26.90 | 24.00 | 25.60 | 23.70 | 21.00 | 21.70 | 20.40 | 24.90 | 20.20 | 22.90 | 19.60 | 20.50 | 19.10 | 19.25 | 19.00 | 22.10 | 19.20 | 21.50 | 19.80 | 25.50 |
|  | 18.60 | 17.80 | 19.50 | 28.60 | 19.60 | 22.90 | 20.05 | 22.80 | 24.30 | 19.80 | 18.38 | 18.95 | 23.13 | 20.97 | 22.31 | 21.16 | 21.64 | 26.89 | 22.39 | 19.97 |
|  | 19.70 | 20.25 | 18.60 | 19.40 | 20.70 | 19.00 | 20.90 | 19.90 | 19.80 | 16.20 | 21.79 | 24.48 | 20.25 | 20.25 | 26.79 | 20.33 | 19.69 | 20.24 | 20.29 | 18.84 |
|  | 21.40 | 18.40 | 21.90 | 23.20 | 23.50 | 18.90 | 19.40 | 20.90 | 19.80 | 20.20 | 19.80 | 19.60 | 18.90 | 21.40 | 20.20 | 20.00 | 19.80 | 19.90 | 20.80 | 21.30 |
| Length (cm) | 4.90 | 4.80 | 5.10 | 4.80 | 4.70 | 5.10 | 4.60 | 4.80 | 4.60 | 4.90 | 4.90 | 4.90 | 5.20 | 4.80 | 5.00 | 4.80 | 5.00 | 4.80 | 5.00 | 5.30 |
|  | 5.20 | 5.00 | 5.00 | 4.80 | 5.10 | 5.00 | 5.30 | 5.00 | 5.20 | 5.20 | 4.70 | 5.10 | 5.10 | 4.90 | 5.00 | 5.00 | 5.00 | 5.00 | 4.80 | 4.80 |
|  | 4.70 | 4.90 | 4.90 | 4.70 | 4.90 | 5.30 | 4.90 | 4.80 | 5.00 | 4.80 | 5.20 | 5.00 | 5.10 | 5.00 | 4.80 | 5.10 | 5.10 | 5.00 | 5.10 | 5.00 |
|  | 5.10 | 5.00 | 5.00 | 5.10 | 5.00 | 5.30 | 5.10 | 5.10 | 5.40 | 5.30 | 4.80 | 4.90 | 5.00 | 5.30 | 4.90 | 4.80 | 4.80 | 4.80 | 4.90 | 4.90 |
|  | 5.10 | 5.20 | 5.10 | 5.20 | 5.00 | 5.00 | 5.20 | 5.20 | 4.70 | 5.00 | 5.20 | 5.30 | 5.00 | 5.10 | 5.10 | 5.00 | 4.80 | 5.10 | 5.10 | 5.30 |
|  | 5.00 | 5.00 | 5.00 | 5.30 | 5.40 | 5.10 | 5.30 | 4.90 | 5.10 | 5.00 | 4.80 | 4.80 | 4.90 | 4.80 | 5.10 | 5.00 | 4.80 | 5.10 | 4.90 | 5.20 |
|  | 5.10 | 5.10 | 5.00 | 5.20 | 5.10 | 5.40 | 5.30 | 5.00 | 5.50 | 5.10 | 4.90 | 4.90 | 4.70 | 4.90 | 5.10 | 5.10 | 4.60 | 5.00 | 4.80 | 5.00 |
|  | 5.10 | 5.00 | 5.50 | 5.40 | 5.30 | 5.20 | 5.30 | 5.50 | 4.90 | 5.10 | 5.00 | 4.80 | 4.90 | 5.00 | 5.20 | 5.10 | 5.20 | 4.80 | 4.90 | 5.10 |
|  | 4.80 | 5.00 | 5.20 | 5.30 | 5.60 | 4.80 | 4.90 | 4.70 | 4.90 | 4.90 | 5.20 | 5.00 | 5.30 | 4.80 | 5.10 | 4.90 | 5.00 | 5.30 | 5.10 | 5.30 |
|  | 5.50 | 5.30 | 5.10 | 5.10 | 5.20 | 4.90 | 5.40 | 5.30 | 5.20 | 5.10 | 5.10 | 5.60 | 5.50 | 5.00 | 4.90 | 5.30 | 5.10 | 5.30 | 5.50 | 5.00 |
| Diameter (cm) | 2.50 | 2.40 | 2.70 | 2.50 | 2.60 | 2.70 | 2.60 | 2.50 | 2.40 | 2.50 | 2.80 | 2.60 | 2.70 | 2.80 | 2.70 | 2.80 | 2.60 | 2.70 | 2.70 | 2.80 |
|  | 2.50 | 2.80 | 2.60 | 2.40 | 2.50 | 2.70 | 2.50 | 2.60 | 2.40 | 2.70 | 2.70 | 2.40 | 2.50 | 2.80 | 2.70 | 2.80 | 2.80 | 2.70 | 2.80 | 2.70 |
|  | 2.60 | 2.50 | 2.60 | 2.70 | 2.40 | 2.80 | 2.70 | 2.50 | 2.80 | 2.40 | 2.50 | 2.60 | 2.50 | 2.40 | 2.50 | 2.50 | 2.40 | 2.70 | 2.60 | 2.50 |
|  | 2.40 | 2.70 | 2.50 | 2.70 | 2.60 | 2.80 | 2.50 | 2.80 | 2.60 | 2.60 | 2.50 | 2.60 | 2.50 | 2.70 | 2.60 | 2.50 | 2.40 | 2.60 | 2.40 | 2.60 |
|  | 2.80 | 2.40 | 2.40 | 2.60 | 2.40 | 2.50 | 2.70 | 2.50 | 2.60 | 2.60 | 2.50 | 2.60 | 2.60 | 2.40 | 2.50 | 2.60 | 2.50 | 2.60 | 2.50 | 2.60 |
|  | 2.60 | 2.70 | 2.60 | 2.50 | 2.80 | 2.60 | 2.70 | 2.80 | 2.70 | 2.70 | 2.60 | 2.70 | 2.50 | 2.80 | 2.40 | 2.50 | 2.60 | 2.50 | 2.40 | 2.50 |
|  | 2.60 | 2.80 | 2.60 | 2.90 | 2.70 | 2.70 | 2.70 | 2.70 | 2.50 | 2.80 | 2.50 | 2.60 | 2.80 | 2.60 | 2.70 | 2.80 | 2.60 | 2.80 | 2.60 | 2.60 |
|  | 2.70 | 2.70 | 2.80 | 2.80 | 2.50 | 2.80 | 2.70 | 2.70 | 2.60 | 2.50 | 2.40 | 2.40 | 2.50 | 2.60 | 2.80 | 2.50 | 2.60 | 2.60 | 2.70 | 2.80 |
|  | 2.80 | 2.40 | 2.70 | 2.60 | 2.50 | 2.60 | 2.60 | 2.70 | 2.50 | 2.60 | 2.80 | 2.80 | 2.50 | 2.90 | 2.70 | 2.70 | 2.80 | 2.70 | 2.80 | 2.70 |
|  | 2.60 | 2.50 | 2.70 | 2.60 | 2.90 | 2.70 | 2.60 | 2.40 | 2.60 | 2.20 | 2.60 | 2.80 | 2.70 | 2.50 | 2.60 | 2.70 | 2.80 | 2.90 | 2.60 | 2.80 |
| Length/diameter (cm) | 1.96 | 2.00 | 1.89 | 1.92 | 1.81 | 1.89 | 1.77 | 1.92 | 1.92 | 1.96 | 1.75 | 1.88 | 1.93 | 1.71 | 1.85 | 1.71 | 1.92 | 1.78 | 1.85 | 1.89 |
|  | 2.08 | 1.79 | 1.92 | 2.00 | 2.04 | 1.85 | 2.12 | 1.92 | 2.17 | 1.93 | 1.74 | 2.13 | 2.04 | 1.75 | 1.85 | 1.79 | 1.79 | 1.85 | 1.71 | 1.78 |
|  | 1.81 | 1.96 | 1.88 | 1.74 | 2.04 | 1.89 | 1.81 | 1.92 | 1.79 | 2.00 | 2.08 | 1.92 | 2.04 | 2.08 | 1.92 | 2.04 | 2.13 | 1.85 | 1.96 | 2.00 |
|  | 2.13 | 1.85 | 2.00 | 1.89 | 1.92 | 1.89 | 2.04 | 1.82 | 2.08 | 2.04 | 1.92 | 1.88 | 2.00 | 1.96 | 1.88 | 1.92 | 2.00 | 1.85 | 2.04 | 1.88 |
|  | 1.82 | 2.17 | 2.13 | 2.00 | 2.08 | 2.00 | 1.93 | 2.08 | 1.81 | 1.92 | 2.08 | 2.04 | 1.92 | 2.13 | 2.04 | 1.92 | 1.92 | 1.96 | 2.04 | 2.04 |
|  | 1.92 | 1.85 | 1.92 | 2.12 | 1.93 | 1.96 | 1.96 | 1.75 | 1.89 | 1.85 | 1.85 | 1.78 | 1.96 | 1.71 | 2.13 | 2.00 | 1.85 | 2.04 | 2.04 | 2.08 |
|  | 1.96 | 1.82 | 1.92 | 1.79 | 1.89 | 2.00 | 1.96 | 1.85 | 2.20 | 1.82 | 1.96 | 1.88 | 1.68 | 1.88 | 1.89 | 1.82 | 1.77 | 1.79 | 1.85 | 1.92 |
|  | 1.89 | 1.85 | 1.96 | 1.93 | 2.12 | 1.86 | 1.96 | 2.04 | 1.88 | 2.04 | 2.08 | 2.00 | 1.96 | 1.92 | 1.86 | 2.04 | 2.00 | 1.85 | 1.81 | 1.82 |
|  | 1.71 | 2.08 | 1.93 | 2.04 | 2.24 | 1.85 | 1.88 | 1.74 | 1.96 | 1.88 | 1.86 | 1.79 | 2.12 | 1.66 | 1.89 | 1.81 | 1.79 | 1.96 | 1.82 | 1.96 |
|  | 2.12 | 2.12 | 1.89 | 1.96 | 1.79 | 1.81 | 2.08 | 2.21 | 2.00 | 2.32 | 1.96 | 2.00 | 2.04 | 2.00 | 1.88 | 1.96 | 1.82 | 1.83 | 2.12 | 1.79 |

Raw data to Table 1: Physical characteristic of date seed Medjool cultivar.

| Component | R1 | R2 | R3 | R4 | R5 | R6 | R7 | R8 | R9 | R10 | R11 | R12 | R12 | R4 | R15 | R16 | R17 | R18 | R19 | R20 |
| --- | --- | --- | --- | --- | --- | --- | --- | --- | --- | --- | --- | --- | --- | --- | --- | --- | --- | --- | --- | --- |
| Weight (g) | 1.20 | 1.30 | 1.20 | 1.30 | 1.20 | 1.40 | 1.30 | 1.40 | 1.30 | 1.20 | 1.40 | 1.30 | 1.40 | 1.40 | 1.40 | 1.30 | 1.20 | 1.30 | 1.30 | 1.20 |
|  | 1.30 | 1.30 | 1.40 | 1.30 | 1.20 | 1.40 | 1.30 | 1.40 | 1.20 | 1.40 | 1.40 | 1.20 | 1.30 | 1.40 | 1.30 | 1.30 | 1.40 | 1.40 | 1.30 | 1.40 |
|  | 1.20 | 1.40 | 1.20 | 1.30 | 1.40 | 1.40 | 1.20 | 1.30 | 1.20 | 1.20 | 1.30 | 1.40 | 1.40 | 1.20 | 1.40 | 1.40 | 1.40 | 1.20 | 1.40 | 1.20 |
|  | 1.40 | 1.20 | 1.20 | 1.40 | 1.30 | 1.40 | 1.20 | 1.30 | 1.30 | 1.30 | 1.20 | 1.30 | 1.20 | 1.40 | 1.20 | 1.40 | 1.30 | 1.40 | 1.30 | 1.20 |
|  | 1.40 | 1.20 | 1.40 | 1.30 | 1.20 | 1.30 | 1.40 | 1.40 | 1.30 | 1.40 | 1.30 | 1.20 | 1.40 | 1.20 | 1.40 | 1.20 | 1.30 | 1.30 | 1.40 | 1.40 |
|  | 1.30 | 1.40 | 1.20 | 1.40 | 1.30 | 1.40 | 1.30 | 1.20 | 1.30 | 1.30 | 1.40 | 1.40 | 1.40 | 1.30 | 1.30 | 1.40 | 1.20 | 1.30 | 1.40 | 1.40 |
|  | 1.40 | 1.20 | 1.40 | 1.40 | 1.20 | 1.40 | 1.30 | 1.20 | 1.30 | 1.40 | 1.20 | 1.30 | 1.40 | 1.30 | 1.30 | 1.40 | 1.30 | 1.40 | 1.20 | 1.40 |
|  | 1.40 | 1.30 | 1.20 | 1.40 | 1.20 | 1.30 | 1.20 | 1.40 | 1.20 | 1.30 | 1.40 | 1.40 | 1.30 | 1.40 | 1.30 | 1.40 | 1.40 | 1.30 | 1.20 | 1.40 |
|  | 1.30 | 1.20 | 1.40 | 1.30 | 1.40 | 1.20 | 1.30 | 1.20 | 1.30 | 1.20 | 1.40 | 1.20 | 1.30 | 1.40 | 1.30 | 1.40 | 1.30 | 1.40 | 1.40 | 1.20 |
|  | 1.20 | 1.40 | 1.20 | 1.30 | 1.20 | 1.20 | 1.40 | 1.40 | 1.30 | 1.40 | 1.30 | 1.30 | 1.40 | 1.40 | 1.30 | 1.30 | 1.40 | 1.30 | 1.40 | 1.40 |
| Length (cm) | 2.50 | 2.60 | 2.60 | 2.50 | 2.40 | 2.60 | 2.70 | 2.40 | 2.50 | 2.50 | 2.70 | 2.60 | 2.60 | 2.80 | 2.80 | 2.70 | 2.60 | 2.70 | 2.50 | 2.80 |
|  | 2.40 | 2.70 | 2.60 | 2.50 | 2.40 | 2.50 | 2.50 | 2.60 | 2.70 | 2.70 | 2.80 | 2.70 | 2.80 | 2.50 | 2.70 | 2.60 | 2.70 | 2.70 | 2.80 | 2.60 |
|  | 2.40 | 2.50 | 2.40 | 2.80 | 2.70 | 2.70 | 2.50 | 2.40 | 2.40 | 2.80 | 2.40 | 2.60 | 2.50 | 2.50 | 2.60 | 2.80 | 2.60 | 2.50 | 2.70 | 2.80 |
|  | 2.50 | 2.60 | 2.30 | 2.30 | 2.60 | 2.50 | 2.30 | 2.40 | 2.50 | 2.40 | 2.50 | 2.40 | 2.40 | 2.50 | 2.60 | 2.40 | 2.40 | 2.40 | 2.60 | 2.50 |
|  | 2.40 | 2.70 | 2.80 | 2.60 | 2.70 | 2.70 | 2.80 | 2.50 | 2.60 | 2.70 | 2.70 | 2.60 | 2.70 | 2.60 | 2.60 | 2.70 | 2.50 | 2.80 | 2.60 | 2.80 |
|  | 2.80 | 2.60 | 2.70 | 2.50 | 2.50 | 2.60 | 2.70 | 2.60 | 2.50 | 2.50 | 2.80 | 2.60 | 2.70 | 2.60 | 2.60 | 2.60 | 2.50 | 2.60 | 2.50 | 2.50 |
|  | 2.50 | 2.70 | 2.50 | 2.50 | 2.40 | 2.70 | 2.60 | 2.30 | 2.60 | 2.60 | 2.40 | 2.70 | 2.60 | 2.40 | 2.60 | 2.50 | 2.70 | 2.60 | 2.50 | 2.40 |
|  | 2.60 | 2.70 | 2.50 | 2.60 | 2.50 | 2.60 | 2.50 | 2.50 | 2.70 | 2.70 | 2.50 | 2.40 | 2.60 | 2.40 | 2.50 | 2.50 | 2.60 | 2.40 | 2.60 | 2.60 |
|  | 2.60 | 2.70 | 2.50 | 2.50 | 2.70 | 2.50 | 2.70 | 2.60 | 2.50 | 2.60 | 2.70 | 2.50 | 2.70 | 2.50 | 2.60 | 2.50 | 2.60 | 2.60 | 2.40 | 2.60 |
|  | 2.60 | 2.80 | 2.70 | 2.80 | 2.50 | 2.70 | 2.60 | 2.70 | 2.60 | 2.70 | 2.40 | 2.70 | 2.80 | 2.70 | 2.60 | 2.70 | 2.70 | 2.60 | 2.70 | 2.60 |
| Diameter (cm) | 0.80 | 0.90 | 0.85 | 0.80 | 0.85 | 0.85 | 0.90 | 0.85 | 0.85 | 0.85 | 0.85 | 0.90 | 0.85 | 0.80 | 0.85 | 0.85 | 0.90 | 0.85 | 0.90 | 0.90 |
|  | 0.80 | 0.90 | 0.85 | 0.85 | 0.80 | 0.85 | 0.85 | 0.90 | 0.85 | 0.90 | 0.80 | 0.85 | 0.85 | 0.90 | 0.80 | 0.90 | 0.90 | 0.85 | 0.90 | 0.85 |
|  | 0.85 | 0.80 | 0.80 | 0.85 | 0.80 | 0.90 | 0.80 | 0.85 | 0.80 | 0.90 | 0.85 | 0.90 | 0.90 | 0.90 | 0.85 | 0.90 | 0.90 | 0.85 | 0.85 | 0.90 |
|  | 0.90 | 0.80 | 0.90 | 0.85 | 0.90 | 0.85 | 0.80 | 0.85 | 0.90 | 0.80 | 0.80 | 0.90 | 0.90 | 0.90 | 0.85 | 0.85 | 0.90 | 0.95 | 0.85 | 0.90 |
|  | 0.85 | 0.90 | 0.90 | 0.80 | 0.90 | 0.90 | 0.90 | 0.85 | 0.85 | 0.90 | 0.80 | 0.90 | 0.85 | 0.80 | 0.85 | 0.90 | 0.85 | 0.90 | 0.85 | 0.90 |
|  | 0.90 | 0.90 | 0.90 | 0.85 | 0.90 | 0.85 | 0.90 | 0.90 | 0.90 | 0.90 | 0.90 | 0.85 | 0.90 | 0.95 | 0.85 | 0.90 | 0.90 | 0.90 | 0.85 | 0.85 |
|  | 0.85 | 0.90 | 0.80 | 0.90 | 0.90 | 0.85 | 0.90 | 0.90 | 0.85 | 0.90 | 0.85 | 0.90 | 0.90 | 0.80 | 0.90 | 0.90 | 0.85 | 0.90 | 0.90 | 0.90 |
|  | 0.90 | 0.85 | 0.90 | 0.90 | 0.80 | 0.90 | 0.90 | 0.90 | 0.80 | 0.90 | 0.80 | 0.90 | 0.80 | 0.90 | 0.90 | 0.85 | 0.85 | 0.90 | 0.85 | 0.90 |
|  | 0.90 | 0.85 | 0.80 | 0.90 | 0.85 | 0.90 | 0.85 | 0.90 | 0.85 | 0.85 | 0.90 | 0.90 | 0.85 | 0.80 | 0.80 | 0.90 | 0.85 | 0.90 | 0.90 | 0.85 |
|  | 0.85 | 0.90 | 0.85 | 0.90 | 0.85 | 0.90 | 0.90 | 0.90 | 0.90 | 0.90 | 0.85 | 0.90 | 0.90 | 0.80 | 0.85 | 0.90 | 0.85 | 0.80 | 0.85 | 0.85 |
| Length/diameter (cm) | 3.13 | 2.89 | 3.06 | 3.13 | 2.82 | 3.06 | 3.00 | 2.82 | 2.94 | 2.94 | 3.18 | 2.89 | 3.06 | 3.50 | 3.29 | 3.18 | 2.89 | 3.18 | 2.78 | 3.11 |
|  | 3.00 | 3.00 | 3.06 | 2.94 | 3.00 | 2.94 | 2.94 | 2.89 | 3.18 | 3.00 | 3.50 | 3.18 | 3.29 | 2.78 | 3.38 | 2.89 | 3.00 | 3.18 | 3.11 | 3.06 |
|  | 2.82 | 3.13 | 3.00 | 3.29 | 3.38 | 3.00 | 3.13 | 2.82 | 3.00 | 3.11 | 2.82 | 2.89 | 2.78 | 2.78 | 3.06 | 3.11 | 2.89 | 2.94 | 3.18 | 3.11 |
|  | 2.78 | 3.25 | 2.56 | 2.71 | 2.89 | 2.94 | 2.88 | 2.82 | 2.78 | 3.00 | 2.67 | 2.67 | 2.67 | 2.78 | 3.06 | 2.82 | 2.67 | 2.53 | 3.06 | 2.78 |
|  | 2.82 | 3.00 | 3.11 | 3.25 | 3.00 | 3.00 | 3.11 | 2.94 | 3.06 | 3.00 | 3.38 | 2.89 | 3.18 | 3.25 | 3.06 | 3.00 | 2.94 | 3.11 | 3.06 | 3.11 |
|  | 3.11 | 2.89 | 3.00 | 2.94 | 2.78 | 3.06 | 3.00 | 2.89 | 2.78 | 2.78 | 3.11 | 3.06 | 3.00 | 2.74 | 3.06 | 2.89 | 2.78 | 2.89 | 2.94 | 2.94 |
|  | 2.94 | 3.00 | 3.13 | 2.78 | 2.67 | 3.18 | 2.89 | 2.56 | 3.06 | 2.89 | 2.82 | 3.00 | 2.89 | 3.00 | 2.89 | 2.78 | 3.18 | 2.89 | 2.78 | 2.67 |
|  | 2.89 | 3.18 | 2.78 | 2.89 | 3.13 | 2.89 | 2.78 | 2.78 | 3.38 | 3.00 | 3.13 | 2.67 | 3.25 | 2.67 | 2.78 | 2.94 | 3.06 | 2.67 | 3.06 | 2.89 |
|  | 2.89 | 3.18 | 3.13 | 2.78 | 3.18 | 2.78 | 2.89 | 2.89 | 2.94 | 3.06 | 3.00 | 2.78 | 3.18 | 3.13 | 3.25 | 2.78 | 3.06 | 2.89 | 2.67 | 3.06 |
|  | 3.06 | 3.11 | 3.18 | 3.11 | 2.94 | 3.00 | 2.89 | 3.00 | 2.89 | 3.00 | 2.82 | 3.00 | 3.11 | 3.38 | 3.06 | 3.00 | 3.18 | 3.25 | 3.18 | 3.06 |

Raw data to Table 2: Proximate composition of date pulp Medjool cultivar.

| **Component** | **Sample 1** | | | **Sample 2** | | | **Sample 3** | | | **Sample 4** | | | **Sample 5** | | |
| --- | --- | --- | --- | --- | --- | --- | --- | --- | --- | --- | --- | --- | --- | --- | --- |
|  | R1 | R2 | R3 | R1 | R2 | R3 | R1 | R2 | R3 | R1 | R2 | R3 | R1 | R2 | R3 |
| Moisture | 24.72 | 25.98 | 25.50 | 27.25 | 28.28 | 28.25 | 26.87 | 26.48 | 24.57 | 24.38 | 25.72 | 23.33 | 24.6 | 25.82 | 25.39 |
| Protein | 3.06 | 3.15 | 3.14 | 3.33 | 3.25 | 3.03 | 3.35 | 3.39 | 3.25 | 2.92 | 2.85 | 2.79 | 3.47 | 3.12 | 3.05 |
| Lipids | 0.78 | 0.74 | 0.76 | 0.68 | 0.71 | 0.72 | 0.81 | 0.82 | 0.79 | 0.74 | 0.75 | 0.77 | 0.74 | 0.76 | 0.75 |
| Fiber | 6.86 | 5.18 | 7.14 | 6.58 | 7.90 | 5.06 | 8.15 | 7.80 | 5.50 | 7.29 | 8.56 | 6.02 | 4.36 | 4.96 | 4.48 |
| Ash | 2.43 | 2.59 | 2.75 | 2.71 | 2.75 | 2.66 | 2.73 | 2.67 | 2.45 | 2.68 | 2.65 | 2.69 | 2.48 | 2.55 | 2.45 |
| Total Carbohydrates | 69.01 | 67.54 | 67.85 | 66.03 | 65.01 | 65.34 | 66.24 | 66.64 | 68.94 | 69.28 | 68.03 | 70.42 | 68.71 | 67.75 | 68.36 |

Raw data to Table 2: Proximate composition of date seed Medjool cultivar (%, g/100g)

| **Component** | **Sample 1** | | | **Sample 2** | | | **Sample 3** | | | **Sample 4** | | | **Sample 5** | | |
| --- | --- | --- | --- | --- | --- | --- | --- | --- | --- | --- | --- | --- | --- | --- | --- |
|  | R1 | R2 | R3 | R1 | R2 | R3 | R1 | R2 | R3 | R1 | R2 | R3 | R1 | R2 | R3 |
| Moisture | 2.95 | 2.58 | 2.69 | 1.72 | 1.81 | 1.54 | 1.77 | 1.71 | 1.50 | 1.25 | 1.40 | 1.35 | 2.76 | 2.81 | 2.99 |
| Protein | 4.83 | 4.95 | 5.18 | 5.35 | 5.42 | 5.67 | 4.48 | 4.68 | 4.33 | 4.60 | 4.37 | 4.34 | 4.87 | 4.75 | 4.85 |
| Lipids | 10.02 | 9.95 | 10.06 | 9.95 | 9.75 | 9.92 | 9.95 | 9.98 | 9.92 | 9.95 | 9.96 | 9.80 | 9.98 | 9.95 | 9.90 |
| Fiber | 64.41 | 65.94 | 64.45 | 71.30 | 70.49 | 71.44 | 61.51 | 62.75 | 62.02 | 69.03 | 70.39 | 70.30 | 66.30 | 66.2 | 65.35 |
| Ash | 1.22 | 1.20 | 1.24 | 1.31 | 1.28 | 1.26 | 1.24 | 1.27 | 1.26 | 1.25 | 1.20 | 1.18 | 1.20 | 1.18 | 1.24 |
| Total Carbohydrates | 80.98 | 81.32 | 80.83 | 81.67 | 81.74 | 81.61 | 82.56 | 82.36 | 82.99 | 82.95 | 83.07 | 83.33 | 81.19 | 81.31 | 81.02 |

Raw data to Table 3: The content of total soluble and insoluble solids, total acidity and pH of pulp of date Medjool cultivar.

| **Component** | **Sample 1** | | | **Sample 2** | | | **Sample 3** | | | **Sample 4** | | | **Sample 5** | | |
| --- | --- | --- | --- | --- | --- | --- | --- | --- | --- | --- | --- | --- | --- | --- | --- |
|  | R1 | R2 | R3 | R1 | R2 | R3 | R1 | R2 | R3 | R1 | R2 | R3 | R1 | R2 | R3 |
| Total soluble solids | 72.21 | 72.25 | 70.10 | 64.89 | 65.30 | 63.76 | 73.10 | 73.84 | 71.90 | 69.43 | 72.77 | 62.67 | 64.41 | 63.65 | 63.49 |
| Insoluble solids | 17.68 | 16.23 | 18.18 | 15.67 | 16.47 | 14.58 | 16.02 | 13.72 | 18.02 | 16.62 | 16.71 | 15.16 | 13.65 | 14.03 | 13.78 |
| Total acidity | 0.07 | 0.06 | 0.08 | 0.08 | 0.09 | 0.07 | 0.07 | 0.06 | 0.09 | 0.06 | 0.08 | 0.08 | 0.07 | 0.09 | 0.08 |
| pH | 6.79 | 6.85 | 6.82 | 6.86 | 6.85 | 6.81 | 6.94 | 6.79 | 6.83 | 6.76 | 6.86 | 6.82 | 6.91 | 6.93 | 6.92 |

Raw data to Table 3: The content of total soluble and insoluble solids, total acidity and pH of seed of date Medjool cultivar.

| **Component** | **Sample 1** | | | **Sample 2** | | | **Sample 3** | | | **Sample 4** | | | **Sample 5** | | |
| --- | --- | --- | --- | --- | --- | --- | --- | --- | --- | --- | --- | --- | --- | --- | --- |
|  | R1 | R2 | R3 | R1 | R2 | R3 | R1 | R2 | R3 | R1 | R2 | R3 | R1 | R2 | R3 |
| Total soluble solids | 3.98 | 3.98 | 4.00 | 3.99 | 3.99 | 4.00 | 8.00 | 8.00 | 8.00 | 6.00 | 6.00 | 6.00 | 4.00 | 4.00 | 4.00 |
| Insoluble solids | 96.0 | 98.0 | 98.0 | 96.0 | 98.0 | 98.0 | 94.0 | 92.0 | 92.0 | 96.0 | 98.0 | 96.0 | 98.0 | 98.0 | 98.0 |
| Total acidity | 0.05 | 0.04 | 0.04 | 0.03 | 0.05 | 0.04 | 0.04 | 0.05 | 0.04 | 0.05 | 0.05 | 0.04 | 0.05 | 0.05 | 0.04 |
| pH | 7.00 | 7.00 | 6.98 | 6.89 | 7.00 | 6.98 | 6.87 | 7.00 | 7.00 | 6.96 | 7.00 | 6.97 | 7.00 | 7.00 | 7.00 |

Raw data to Table 4: Sugar content of date pulp Medjool cultivar.

| **Component** | **Sample 1** | | | **Sample 2** | | | **Sample 3** | | | **Sample 4** | | | **Sample 5** | | |
| --- | --- | --- | --- | --- | --- | --- | --- | --- | --- | --- | --- | --- | --- | --- | --- |
|  | R1 | R2 | R3 | R1 | R2 | R3 | R1 | R2 | R3 | R1 | R2 | R3 | R1 | R2 | R3 |
| Total sugars | 78.08 | 74.10 | 80.00 | 77.57 | 79.29 | 78.13 | 72.56 | 73.33 | 73.75 | 71.10 | 77.17 | 71.21 | 72.50 | 75.45 | 76.01 |
| Reducing sugars | 71.03 | 70.89 | 69.45 | 74.38 | 72.69 | 73.96 | 68.41 | 67.63 | 66.57 | 70.42 | 72.03 | 73.91 | 65.22 | 66.51 | 70.25 |
| Sucrose | 5.10 | 5.08 | 5.02 | 5.14 | 5.12 | 5.03 | 5.12 | 5.15 | 5.09 | 4.94 | 4.90 | 4.96 | 5.07 | 5.00 | 5.12 |
| Glucose | 38.75 | 37.83 | 37.98 | 38.75 | 37.95 | 37.78 | 33.00 | 34.09 | 34.05 | 37.92 | 37.69 | 38.06 | 37.65 | 37.96 | 38.59 |
| Fructuose | 33.83 | 33.98 | 34.97 | 35.02 | 34.59 | 33.61 | 29.80 | 30.96 | 31.77 | 33.49 | 32.71 | 32.23 | 33.86 | 32.68 | 33.99 |
| Energetic value | 342.48 | 325.67 | 350.59 | 340.21 | 347.48 | 342.11 | 320.31 | 323.73 | 324.88 | 312.46 | 337.86 | 312.84 | 319.79 | 331.43 | 333.51 |

Raw data to Table 4: Sugar content of date seed Medjool cultivar.

| **Component** | **Sample 1** | | | **Sample 2** | | | **Sample 3** | | | **Sample 4** | | | **Sample 5** | | |
| --- | --- | --- | --- | --- | --- | --- | --- | --- | --- | --- | --- | --- | --- | --- | --- |
|  | R1 | R2 | R3 | R1 | R2 | R3 | R1 | R2 | R3 | R1 | R2 | R3 | R1 | R2 | R3 |
| Total sugars | 5.89 | 6.05 | 5.76 | 5.75 | 5.97 | 6.03 | 5.56 | 5.36 | 5.50 | 6.20 | 6.12 | 6.26 | 5.62 | 5.89 | 5.93 |
| Reducing sugars | 4.39 | 4.38 | 4.43 | 4.39 | 4.37 | 4.47 | 4.41 | 4.51 | 4.43 | 4.47 | 4.44 | 4.50 | 4.51 | 4.45 | 4.57 |
| Sucrose | 1.50 | 1.67 | 1.33 | 1.36 | 1.60 | 1.56 | 1.15 | 0.85 | 1.07 | 1.73 | 1.68 | 1.76 | 1.11 | 1.44 | 1.36 |
| Glucose | - | - | - | - | - | - | - | - | - | - | - | - | - | - | - |
| Fructuose | - | - | - | - | - | - | - | - | - | - | - | - | - | - | - |
| Energetic value | 121.26 | 121.66 | 121.97 | 121.45 | 120.88 | 123.21 | 118.37 | 118.31 | 117.48 | 121.37 | 120.52 | 119.69 | 119.9 | 120.46 | 120.48 |

Raw data to Table 5: Mineral content of date pulp and seed Medjool cultivar.

| **Component** | **Pulp** | | | **Seed** | | |
| --- | --- | --- | --- | --- | --- | --- |
|  | R1 | R2 | R3 | R1 | R2 | R3 |
| Potassium | 832.19 | 849.23 | 874.55 | 399.97 | 425.87 | 414.21 |
| Magnesium | 136.85 | 143.01 | 149.05 | 39.71 | 35.29 | 32.85 |
| Calcium | 124.33 | 131.74 | 131.49 | 50.03 | 59.01 | 53.8 |
| Phosphorus | 139.43 | 135.41 | 143.37 | 98.12 | 86.80 | 92.54 |
| Sulfur | 108.33 | 111.00 | 110.33 | 162.66 | 137.85 | 153.55 |
| Sodium | 27.35 | 27.73 | 27.87 | 38.32 | 33.16 | 30.75 |
| Silicon | 8.11 | 11.3 | 14.32 | 0.87 | 0.81 | 0.70 |
| Selenium | 5.35 | 5.01 | 5.38 | 3.90 | 4.00 | 4.33 |
| Copper | 1.05 | 1.14 | 0.91 | 0.94 | 0.81 | 0.75 |
| Iron | 0.31 | 0.36 | 0.34 | 1.45 | 1.08 | 1.43 |
| Manganese | 0.41 | 0.46 | 0.43 | 0.79 | 0.63 | 0.91 |
| Zinc | 0.17 | 0.28 | 0.31 | 1.27 | 0.77 | 1.22 |

Raw data to Table 6: Fatty acid compositions of date pulp and seed Medjool cultivar.

| **Component** | **Pulp** | | | **Seed** | | |
| --- | --- | --- | --- | --- | --- | --- |
|  | R1 | R2 | R3 | R1 | R2 | R3 |
| Caprylic (C8:0) | - | - | - | 0.283 | 0.279 | 0.272 |
| Capric (C10:0) | - | - | - | 0.360 | 0.343 | 0.370 |
| Lauric (C12:0) | - | - | - | 17.44 | 17.17 | 17.09 |
| Myristic (C14:0) | - | - | - | 10.86 | 10.44 | 10.86 |
| Palmitic (C16:0) | 6.77 | 6.79 | 6.70 | 10.81 | 10.61 | 10.86 |
| Palmitoleic (C16:1) | - | - | - | 0.067 | 0.066 | 0.066 |
| Margaric (C17:0) | - | - | - | 0.059 | 0.057 | 0.056 |
| Stearic (C18:0) | 3.96 | 3.96 | 4.04 | 4.83 | 4.65 | 4.88 |
| Oleic (C18:1) | 52.08 | 52.67 | 52.26 | 45.43 | 46.69 | 45.63 |
| Vaccenic (C18:1) | 5.43 | 4.61 | 4.36 | - | - | - |
| Linoleic (C18:2) | 30.25 | 30.40 | 31.03 | 9.06 | 9.04 | 9.06 |
| Eicosanoic (C20:0) | 0.64 | 0.65 | 0.66 | 0.47 | 0.42 | 0.45 |
| Eicosenoic (C20:1) | - | - | - | 0.23 | 0.21 | 0.20 |
| Gondoic (C20:1) | 0.89 | 0.92 | 0.96 | - | - | - |

Raw data to Table 7: Total phenolic content and antioxidant activities of date pulp Medjool cultivar.

| **Component** | **Sample 1** | | | **Sample 2** | | | **Sample 3** | | | **Sample 4** | | | **Sample 5** | | |
| --- | --- | --- | --- | --- | --- | --- | --- | --- | --- | --- | --- | --- | --- | --- | --- |
|  | R1 | R2 | R3 | R1 | R2 | R3 | R1 | R2 | R3 | R1 | R2 | R3 | R1 | R2 | R3 |
| Total phenolic content | 1.169 | 1.156 | 1.165 | 1.167 | 1.168 | 1.167 | 1.189 | 1.169 | 1.179 | 1.155 | 1.158 | 1.158 | 1.167 | 1.156 | 1.165 |
| β – Carotene | 69 | 71 | 70 | 71 | 72 | 72 | 67 | 67 | 56 | 65 | 64 | 63 | 56 | 60 | 65 |
| DPPH | 0.097 | 0.053 | 0.057 | 0.091 | 0.091 | 0.091 | 0.093 | 0.094 | 0.093 | 0.060 | 0.063 | 0.069 | 0.074 | 0.073 | 0.078 |
| ABTS | 14.52 | 14.34 | 14.22 | 14.51 | 14.52 | 13.84 | 13.19 | 13.23 | 13.31 | 13.36 | 12.56 | 13.54 | 13.45 | 13.18 | 13.87 |

Raw data to Table 7: Total phenolic content and antioxidant activities of date seed Medjool cultivar.

| **Component** | **Sample 1** | | | **Sample 2** | | | **Sample 3** | | | **Sample 4** | | | **Sample 5** | | |
| --- | --- | --- | --- | --- | --- | --- | --- | --- | --- | --- | --- | --- | --- | --- | --- |
|  | R1 | R2 | R3 | R1 | R2 | R3 | R1 | R2 | R3 | R1 | R2 | R3 | R1 | R2 | R3 |
| Total phenolic content | 13.91 | 13.82 | 13.97 | 13.94 | 14.73 | 13.81 | 13.91 | 13.74 | 13.58 | 12.97 | 12.25 | 12.70 | 14.25 | 14.25 | 14.26 |
| β - Carotene | 39 | 38 | 45 | 48 | 39 | 39 | 55 | 50 | 44 | 56 | 54 | 53 | 61 | 49 | 47 |
| DPPH | 0.0019 | 0.0009 | 0.0014 | 0.0036 | 0.0028 | 0.0101 | 0.0085 | 0.0080 | 0.0118 | 0.0118 | 0.0013 | 0.0012 | 0.0038 | 0.0030 | 0.0026 |
| ABTS | 0.258 | 0.144 | 0.126 | 0.262 | 0.272 | 0.263 | 0.262 | 0.265 | 0.268 | 0.203 | 0.235 | 0.216 | 0.211 | 0.240 | 0.320 |
